# Application of microRNA In Situ Hybridization on Long-term Stored Human Formalin-fixed Paraffin-embedded Brain Samples from Psychiatric Patients

**DOI:** 10.1007/s12035-025-05077-z

**Published:** 2025-05-31

**Authors:** Rolf Søkilde, Erik Kaadt, Lasse Sommer Kristensen, Morten Trillingsgaard Venø, Jørgen Kjems, Jens Randel Nyengaard, Boye Schnack Nielsen, Betina Elfving

**Affiliations:** 1 Experimental and Molecular Psychiatry, Translational Neuropsychiatry Unit, Department of Clinical Medicine, Aarhus University, Palle Juul-Jensens Blvd 11, 8200 Aarhus, Denmark; 2 The Laboratory for Gene-Regulatory Mechanisms in Cancer, Department of Biomedicine, Aarhus University, Høegh-Guldbergs Gade 10, 8000 Aarhus, Denmark; 3 omiics ApS, Åbogade 15, 8200 Aarhus, Denmark; 4 Department of Molecular Biology and Genetics (MBG), Aarhus University, Nørrebrogade 44, 8000 Aarhus, Denmark; 5 Interdisciplinary Nanoscience Center (iNANO), Aarhus University, Gustav Wieds Vej 14, 8000 Aarhus, Denmark; 6 Core Center for Molecular Morphology, Section for Stereology and Microscopy, Department of Clinical Medicine, Aarhus University, Palle Juul-Jensens Blvd 35, 8200 Aarhus, Denmark; 7 Department of Pathology, Aarhus University Hospital, Palle Juul-Jensens Boulevard 99, 8200 Aarhus, Denmark; 8 Department of Cellular Engineering & Disease Modeling, Bioneer A/S, Kogle Alle 2, 2970 Hørsholm, Denmark

**Keywords:** MicroRNA, In situ hybridization, Brain, Psychiatric disorders, FFPE, MiRNAscope

## Abstract

Here, we report that long-term stored human brain samples, formalin-fixed paraffin-embedded (FFPE) from The Brain Collection, University of Southern Denmark, Denmark (https://www.sdu.dk/en/forskning/bridge/the-brain-collection), can be used for in situ hybridization (ISH) analysis of selected microRNAs (miRNAs). The Human Brain Collection consists of brains from 9479 subjects who died at a Danish State Mental Hospital in the period of 1945–1982. In the present study we included tissue specimens from prefrontal cortex (PFC) and hippocampus (HIP) from 163 patients diagnosed with schizophrenia, bipolar disorder, or major depressive disorder. Initially, the Nanostring nCounter platform was used to identify miRNA candidates for ISH analysis using the miRNAscope technology. Based on the Nanostring nCounter quantifications with bulk tissue, we identified and selected 10 miRNAs from PFC (miR-9-5p, miR-29b-3p, miR-30c-5p, miR-124-3p, miR-125b-5p, miR-138-5p, miR-181a-5p, miR-224-5p, miR-302d-3p, and miR-432-5p) and 6 miRNAs from HIP (let-7a-5p, miR-7-5p, miR-124-3p, miR-127-3p, miR-145-5p and miR-149-5p). miRNAscope ISH analysis was then performed with the respective probes on 30 PFC and 30 HIP samples, respectively. In the PFC six miRNAs (miR-9-5p, miR-29b-3p, miR-124-3p, miR-125b-5p, miR-138-5p, and miR-181a-5p) were detected and four (miR-145-5p, let-7a-5p, miR-124-3p and miR-7-5p) in the HIP samples. In both brain regions miR-124-3p was the most abundantly expressed. We conclude, that the combination of the Nanostring nCounter technology and the miRNAscope analysis is a valid approach to study spatial expression of specific miRNAs in these up to 76 years old FFPE blocks. This opens a new avenue of possibilities for studying the underlying epigenetic mechanisms in mental disorders.

**Supplementary Information:**

The online version contains supplementary material available at 10.1007/s12035-025-05077-z.

## Introduction

The Brain Collection, University of Southern Denmark, Denmark (https://www.sdu.dk/en/forskning/bridge/the-brain-collection), consists of brains from 9479 subjects who died at a Danish State Mental Hospital in the period of 1945–1982.

First time the Human Brain Collection was presented internationally was at the BrainNet Europe International Conference on Human Brain Tissue in June 2006 [1]. Today, the collection is one of the largest collections of its kind worldwide. The biobank consists of tissues fixed in formalin and embedded in paraffin, as well as tissues stored in buckets with formalin. Associated histological slides are archived, and medical records are filed for each patient. The presented work is performed on sections from the formalin-fixed paraffin-embedded (FFPE) tissue blocks. Previously, DNA methylation status has been evaluated in these FFPE tissue blocks [2]. The authors found that the DNA quality was inversely correlated with storage time, which affected the usability of the data. In another study, samples from the brain collection were thionin stained to determin the hippocampal volume and the number of neurons and glial cells in patients with major depressive disorder (MDD) and schizophrenia (SCZ) [3]. Recently, morphological alterations of pyramidal cells in cortical layer III of the prefrontal cortex were reported [4]. Hence, it is of great interest to explore, if other modern molecular techniques can be used for investigation of the great resource the Human Brain Collection contains.

MicroRNAs (miRNA) are a class of small non-coding RNAs (20–25 nucleotides), which main function is to post-transcriptionally regulate the levels and translation of target mRNA molecules [5]. This function is performed through the RNA-induced silencing complex (RISC), which identifies the miRNA target site primarily in the 3-UTR region of mRNAs [5–7]. Individual miRNAs usually have many targets and individual mRNAs have many miRNA target sites, constructing complex regulatory networks. The function of individual miRNAs have been investigated and miRNAs have been shown to be key regulators for brain development, as well as to the function of adult cells of the brain [8, 9]. Notably, miR-124-3p is the most abundant miRNA in the human brain, and is a pan-neuronal miRNA that is believed to control neuronal differentiation [10]. miR-124-3p has genetically been associated with MDD, SCZ, and bipolar disorder (BD) in postmortem prefrontal cortex human brain samples [11–13], as well as dysregulated miR-124-3p blood levels has been linked to MDD [11–14].

Successful miRNA expression profiling has previously been reported in older samples and miRNAs are considered more resistant to degradation than longer RNAs like mRNA probably because they are associated with proteins that may shield them from degradation [15]. As limited amounts of FFPE tissue are available from the Human Brain Collection, we aim as a part of the BioPsych project (Identification of BIOmarkers in the human PSYCHiatric brain – focusing on non-coding RNAs and sex differences, https://projects.au.dk/odin/biopsych) to establish a workflow that allows for selection of relevant miRNAs for in situ Hybridization (ISH) analysis. As RNA-seq has been reported challenging using FFPE samples, and the Nanostring technology has been reported to be superior [16], we considered a relatively unbiased profiling of miRNA using the Nanostring nCounter Platform on bulk tissue, followed by ISH. Based on the expression levels of miRNAs from the Nanostring nCounter platform, we here selected 15 unique miRNAs in a screening effort with ISH analysis using the miRNAscope technology in two core brain areas of psychiatric disorders, the prefrontal cortex and hippocampus. MiRNAscope is a relatively newly invented ISH technology based on Z-probe design for RNA detection and branched DNA amplification for signal detection [17], and was chosen here due to expected high specificity and sensitivity, and that the method is available as an automated staining protocol on the Leica Bond RX instrument, thereby potentially improving staining reproducibility [18].

Using this platform, we report that 9 out of 15 selected miRNAs in the ISH screening yielded signal, demonstrating that at least the selected cases from the old human brain collection can be used for miRNA ISH.

## Materials and Methods

### Brain Bank Description of Postmortem Brain Samples from Each Subject

The relevant patient data, type of psychiatric disorder, age, sex, storage time and type of molecular analysis can be seen in supplementary table (ST1).

The individuals were all Scandinavian Caucasians with no history of alcohol and drug misuse, and were collected in accordance with Danish law and with the consent of the Central Denmark Area Health Research Ethics Committees (license number: M-2017–17-17). Two very experienced psychiatrists (AB Bertelsen and R Rosenberg) reassessed and verified the diagnoses by carefully reviewing all medical records and comparing the diagnoses with the modern criteria of DSM-IV (Diagnostic and Statistical Manual of Mental Disorders, 4 th Edition) and ICD-10 criteria (The International Statistical Classification of Diseases and Related Health Problems 10 th Edition). In the present study we included patients diagnosed with SCZ, BD and MDD and samples from prefrontal cortex and hippocampus. The Brain Collection does not include healthy controls.

### RNA Extraction and microRNA Concentration

**Fig. 1:**
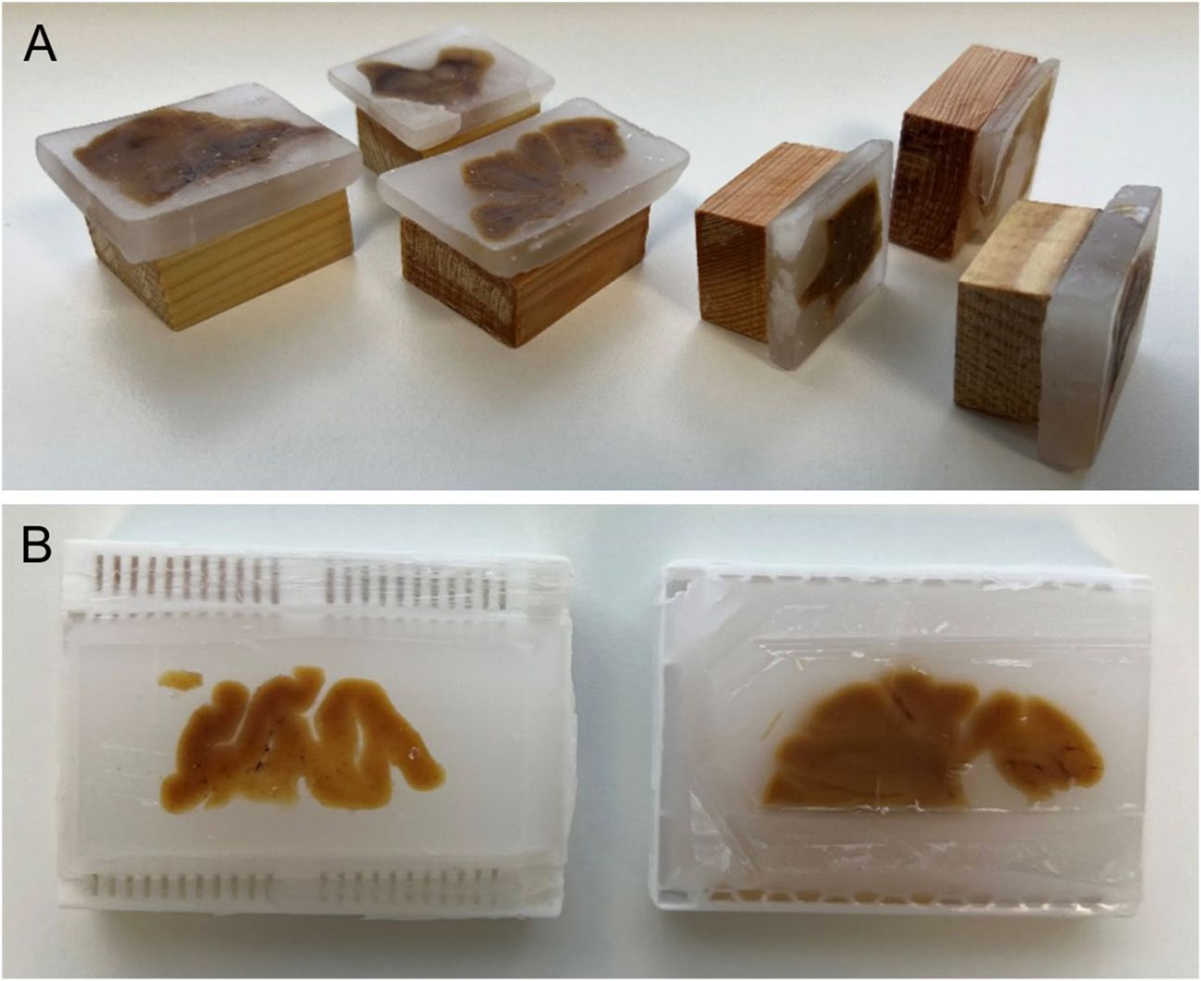
Re-embedding old paraffin blocks. Examples of original paraffin blocks produced from 1945–1982 (**A**). The brain tissue specimens had been fixed and paraffin embedded and mounted onto wooden blocks. For the current study the old paraffin blocks were mounted onto modern plastic cassettes (**B**)

The original FFPE samples were placed on wooden blocks and for processing tissue sections on a modern microtome, the samples were replaced onto modern plastic cassettes (Fig. 1).

Blocks were trimmed and 12 sections of 10 µm were cut. The 12 slices were divided into two separate Eppendorf tubes, with 6 slices in each tube. These were processed as separate samples following the manufacturer's instructions until they were loaded onto the RNeasy MinElute spin column. At this point, the 12 slices were pooled on the same column. The miRNA concentration was measured using the Qubit 3.0 fluorometer (Thermo Fisher Scientific, USA, RRID:SCR_020311) according to the Qubit microRNA Assays Kits protocol.

### NanoString nCounter microRNA Assay

The Human v3b miRNA panel detecting 827 endogenous miRNAs was used for the nCounter assays. All samples were normalized to a Qubit miRNA concentration of 30 ng/µl and analyzed on the nCounter SPRINT platform (nanoString Technologies, USA, RRID:SCR_021712). An overview of the samples and in what part of our study they have been used is given in supplementary Table 1 (ST1).

Mean, standard deviation (SD) and plots of the nanoString data was performed in R Project for Statistical Computing (RRID:SCR_001905). For estmation of the detection of miRNAs we used the nanoString recommended limit of detection (average of negative controls + 2*SD) to find a total of 313 miRNAs were detected in hippocampus and 171 in prefrontal cortex.

### Sectioning for in situ Hybridization

For the miRNA ISH part of the study, 30 patients were selected with representative samples from all three diagnoses and comprising equal proportions of males and females. From the remounted blocks, 5 µm thick sections were cut and mounted on Superfrost Plus glass slides. For a complete overview of overlap of specimens between the studies using the nanoString nCounter and miRNA ISH see ST1.

### Selection of microRNAs for in situ Hybridization

**Fig. 2:**
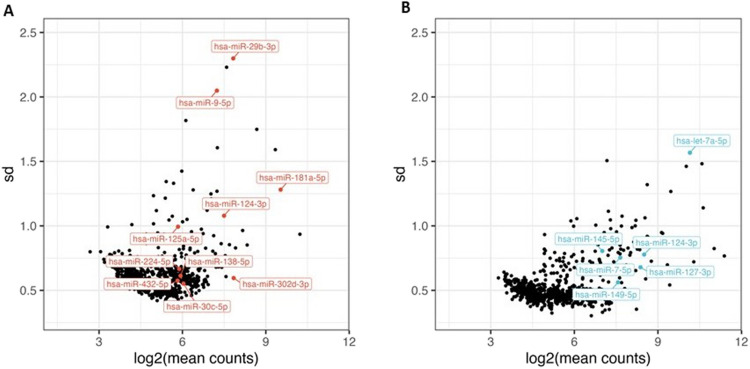
microRNA expression using the Nanostring nCounter technology. miRNAs were extracted from paraffin sections and submitted to quantitative assessment using the nCounter expression panels. The standard deviation (SD) is plotted against the mean count values of the individual miRNAs obtained from the prefrontal cortex (**A**) and hippocampus (**B**) samples. The mean count values show the average expression level and the SD-values indicate the spread of the values in the sample cohort. Among highly expressed miRNAs in prefrontal cortex are miR-181a-5p, miR-124-3p, miR-302 d-3p, miR-29b-3p, and miR-9-5p, and in hippocampus are indicated let-7a-5p, miR-124-3p and miR-127-3p

Based on the results of the nanoString analysis, including expression threshold and compatibility with probe design (ACD bioinformatics team and own observations), we selected 10 miRNA candidates above background expression (miR-124-3p, miR-9-5p, miR-29b-3p, miR-125b-5p, miR-138-5p, miR-432-5p, miR-302 d-3p, miR-224-5p, miR-30c-5p, and miR-181a-5p) to be tested on the human prefrontal cortex samples and 6 candidate miRNAs (miR-124-3p, let-7a-5p, miR-127-3p, miR-145-5p, miR-7-5p, and miR-149-5p) to be tested on the hippocampus samples using miRNAscope technology. For an overview of probes and miRNA identifiers see ST2. miR-124-3p is one of the most abundant miRNAs in the brain and was the only probe submitted to the 2 areas. The selected miRNAs spanned the count intensity range of the nanoString nCounter platform (82–1730 mean counts). The SD of the counts was also explored to get an idea of the general variation in signal intensity in the samples (see Fig. 2). The GC-content of the selected miRNAs varied from 35–55%, which is with the expected ranges for miRNAs [19].

### microRNA in situ Hybridization

miRNA ISH was performed according to the miRNAscope manual (ACD, USA, RRID:SCR_012481). miRNAscope probes were obtained from ACD/Biotechne and are listed in Table ST2. In brief, FFPE sections were prebaked at 60 °C for one hour and immediately placed in a Leica Bond RX instrument (LeicaBiosystems, Germany, Cat no:212821). Initial optimization experiments were conducted, with different lengths of the incubation times in Epitope Retrieval buffer 2 (ER2) (15, 20, 25, 30 min) and protease digestion with miRNAscope 2.5 LS Protease III (15, 20, 25, 30 min). After evaluation of the results the following protocol was used: incubation of the sections in ER2 for 30 min at 95 °C, followed by protease treatment with miRNAscope 2.5 LS Protease III for 30 min at 40 °C. Probe incubation was performed at 37 °C for 2 h, followed by repeated washes and incubation with the 6 amplification steps, before signal detection with the alkaline phosphatase-based signal amplification system. Hematoxylin was used for nuclei detection. Slides were rinsed in running tap water before drying and mounting in EcoMount. To obtain digital whole slides, tissue scanning of the stained slides was performed on a Zeiss Axio Scan Z1 brightfield slide scanner with a 20X objective (Zeiss, RRID:SCR_020927). Representative images were acquired using the Zeiss software. miRNAscope probes to small nuclear RNA (snRNA) U6 and to a random generic nucleotide sequence (called scramble) were included as positive and negative assay controls (ST2).

### Scoring of signal Intensities

For scoring the miR-124-3p staining, the overall staining intensities were judged at low magnification. Since all 30 samples were miR-124-3p-positive, they were scored 1, 2, or 3, where 1 referred to cases with relatively low intensity, cases scored as 2 had medium staining intensity and cases scored as 3 had the highest staining intensity (examples are shown in Fig. 4 and the scores listed in ST3). All other cases stained with any miRNAscope probe were inspected and the staining scored binary (0, 1), 1 for positive signal and 0 for the absence of signal (See supplementary table ST3 and ST4). Tissue folds and other artifacts were often present in the sections. This includes wrinkles, folds, debris, uneven stretching and to a limited extent tissue adherence. Tissue areas with tissue artifacts were avoided and scoring was performed only in areas with representative tissue and signal.

## Results

### Selection of samples for in situ Hybridization

For the miRNA ISH part of the study, 30 patients were selected with representative samples from all three diagnoses and comprising both males and females. The sample characteristics are summarized in Table 1. There was no difference in age at time of death for the patients between the diagnoses. However, the mean age at time of death of the patients in the male group was lower than the females, 57.1 and 66.7 years, respectively (ST5).

**Table 1:** Overview of human brain samples included for each disorder. bipolar disorder (BD), major depressive disorder (MDD) and schizophrenia (SCZ)

Characteristic	BD, *N* = 56	MDD, *N* = 51	SCZ, *N* = 56
Sex			
Female	31	26	29
Male	25	25	27
Median Age at death	63 (56, 72)	62 (50, 71)	63 (56, 68)
Nanostring analysis			
Prefrontal cortex	44	32	41
Hippocampus	38	42	39
*In situ* Hybridization			
Prefrontal cortex	10	10	10
Hippocampus	10	10	10

Median (IQR, 25% and 75%)

### NanoString microRNA Profiling

To identify candidate miRNAs, total RNA including small RNAs was obtained from 12 paraffin section of 10 µm from 117 prefrontal cortex samples and 119 hippocampus samples (Table 1 and ST2). NanoString profiling was performed and 313 miRNAs were detected in the hippocampus samples and 171 miRNAs in the prefrontal cortex samples, and from these we selected ten from prefrontal cortex samples and six from hippocampus samples for miRNAscope localization analysis (Fig. 2). The miRNAs were selected to represent a range of expression levels and identified as significantly differentially expressed in at least one comparison (ST2).

### Test of Pretreatment Conditions for in situ Hybridization

**Fig. 3:**
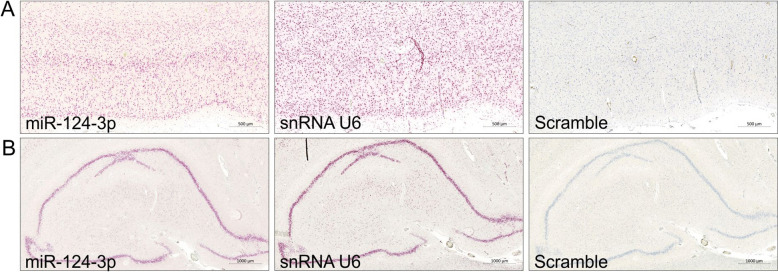
miRNAScope control probes in old formalin-fixed paraffin-embedded (FFPE) blocks. Serial tissue sections were obtained with FFPE samples from prefrontal cortex (panel **A**) and hippocampus (panel **B**) and stained using the automated miRNAScope technology. Representative images show staining with probes for miR-124-3p, snRNA U6 and scramble. High staining intensity (red stain) was obtained with miR-124-3p and snRNA U6 probes in both prefrontal cortex and hippocampus, and virtually no staining with the scramble probe

It was assumed that the old specimens had been extensively fixed and most likely to a varying degree, therefore the pre-treatment conditions, involving ER2 and protease digestion, were initially optimized. Because miR-124-3p is one of the most abundant miRNAs in the brain, the miR-124-3p probe was used for testing pre-treatment conditions in 3 representative specimens from the prefrontal cortex. The ISH signal intensity was found to be generally highest when both the ER2 and protease step were set at 30 min. At these pre-treatment conditions, we also tested the performance of the positive control miRNAscope probe to snRNA U6 and the negative control probe, scramble. The snRNA U6 probe resulted in intense ISH signal with a localization as expected in cell nuclei, and the scramble probe resulted in no staining in the tested samples (Fig. 3).

### miR-124-3p in situ Hybridization Signal Variation in Prefrontal Cortex

**Fig. 4:**
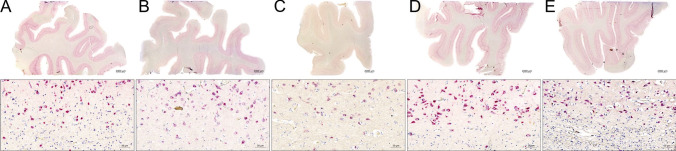
Variation in the performance of old formalin-fixed paraffin-embedded blocks. Tissue sections were obtained from 5 (**A**-**E**) prefrontal cortex samples, and stained for miR-124-3p using automated miRNAScope, then scanned to obtain digital whole slides (upper row) from which a subset region is shown covering grey and white matter interface (lower row). Case **A** and **D** performed with highest staining intensity, **B** and **E** with intermediate staining intensity and **C** with the lowest miR-124-3p staining intensity

**Fig. 5:**
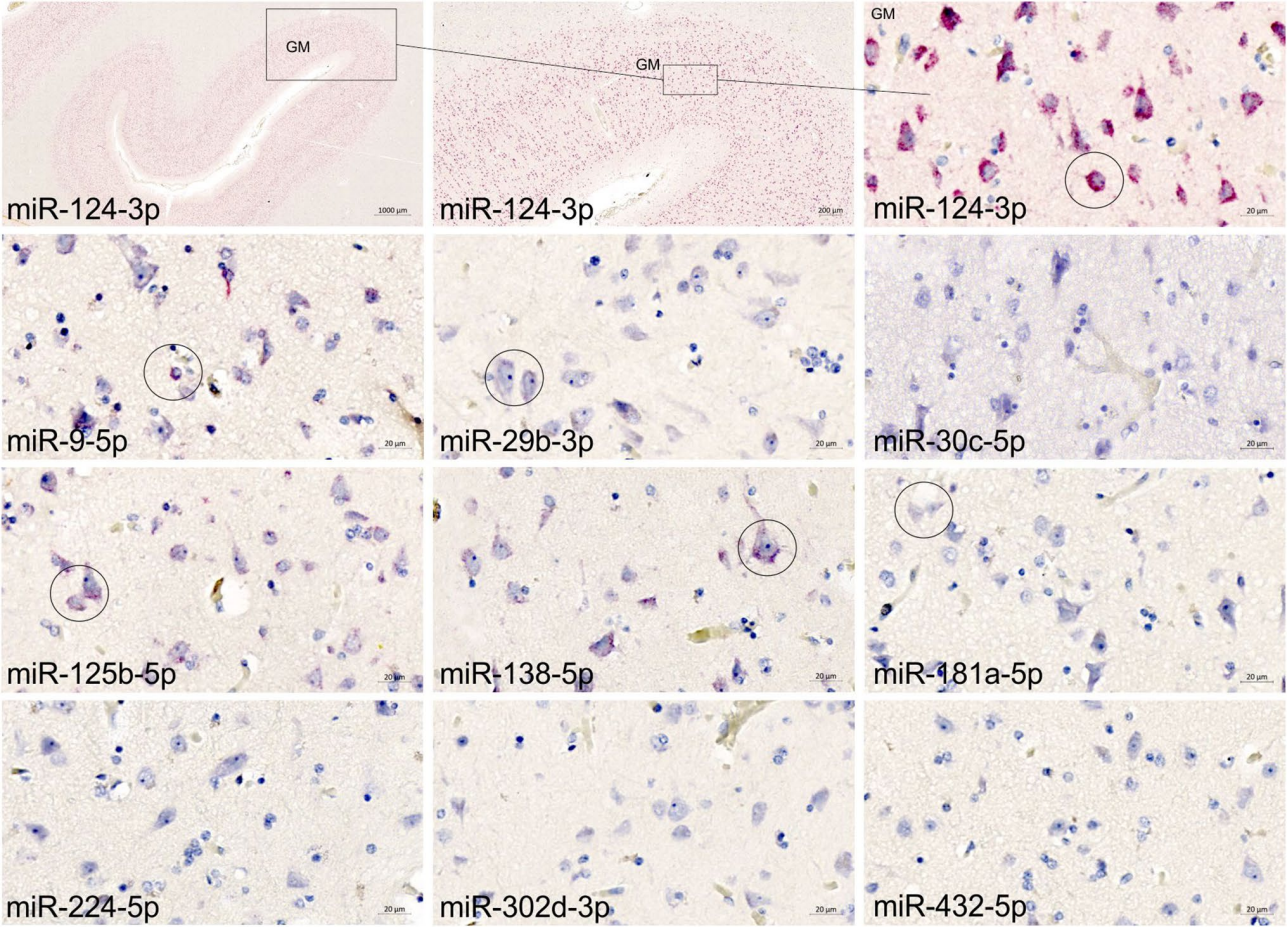
Expression of selected microRNAs in prefrontal cortex, grey matter investigated using in situ Hybridization (ISH). Tissue sections from prefrontal cortex samples were stained for selected miRNAs (Table 2) using automated miRNAScope. Intense ISH signal is seen in the grey matter (GM) with the miR-124-3p probe (upper row) at both high and low magnification, whereas probes for the other miRNAs show a discrete or no signal (and therefore only shown at high magnification). Positive ISH signal (examples indicated by circles) is seen with probes against miR-9-5p, miR-29b-3p, miR-125b-5p, miR-138-5p and miR-181a-5p whereas no ISH signal is seen with probes to miR-30c-5p, miR-224-5p, miR-302 d-3p and miR-432-5p

miRNAscope ISH for miR-124-3p was performed in the 30 prefrontal cortex samples and ISH signal observed in all 30 cases. We scored the 30 samples based on the staining intensity of the whole Sect. 1, 2 or 3, for low, medium and high signal intensity, respectively. Examples are shown in Fig. 4 and the scores listed in ST3. The scores were compared with storage time, but no correlation was found (p-value > 0.05 in a linear model) (Supplementary Fig. 1). The miR-124-3p expression was mainly seen in neurons of the cortical layers of the prefrontal cortex (see Fig. 5). The localization was mainly perinuclear, but signal was also seen in branch structures, most likely neuronal dendrites and in some neurons also overlapping with the nuclei. Samples that scored 1 were considered to be less likely to result in ISH signal with probes targeting the lower expressed microRNAs (see below). Two samples (prefrontal cortex samples from case 32 and 74) performed very poorly.

### microRNA in situ Hybridization with Prefrontal Cortex Samples

The other nine of the ten selected miRNA probes were then tested in the prefrontal cortex samples, and the observations are summarized in Table 2 and ST3. miR-9-5p and miR-29b-3p were tested in all 30 samples from prefrontal cortex. As expected, we also found markedly lower signal for miR-9-5p in the two samples performing poorly with the miR-124-3p probe (32 and 74) compared to the other samples in the cohort, and no ISH signal was seen with the probe to miR-29b-3p. These two samples were therefore not stained with the remaining 7 miRNAscope probes. A manual scoring of all sections was conducted using a binary scale, with present or absent of ISH signal. Positive ISH signal was identified with probes for miR-9-5p, miR-138-5p, miR-29b-3p, miR-125b-5p, and miR-181a-5p (Fig. 5). ISH signal for miR-29b-3p, miR-124-3p and miR-138-5p, was mostly seen in the grey matter, whereas ISH signal for miR-18 1a-5p was mostly seen in the white matter (Supplementary Fig. 2), and ISH signal for miR-9-5p, and miR-125b-5p was seen in both compartments. No signal was found with the probes for miR-302 d-3p, miR-432-5p, miR-224-5p, and miR-30c-5p in any of the samples (Fig. 5).

**Table 2:** Scores from prefrontal cortex with binary staining score (1, 0, NA)

miRNA	Number of sections stained	1	0	NA
miR-9-5p	30	28	2*	0
miR-29b-3p	30	18	12	0
miR-30c-5p	28	0	28	0
miR-124-3p	30	28	2*	0
miR-125b-5p	28	28	0	0
miR-138-5p	28	28	0	0
miR-181a-5p	28	27	0	1
miR-224-5p	28	0	28	0
miR-302 d-3p	28	0	26	4
miR-432-5p	28	0	28	0

* The 2 samples with low quality of miR-124-3p and miR-9-5p stainings, were deselected for further stainings for miRNAs

### microRNA in situ Hybridization with Hippocampus Samples

**Fig. 6:**
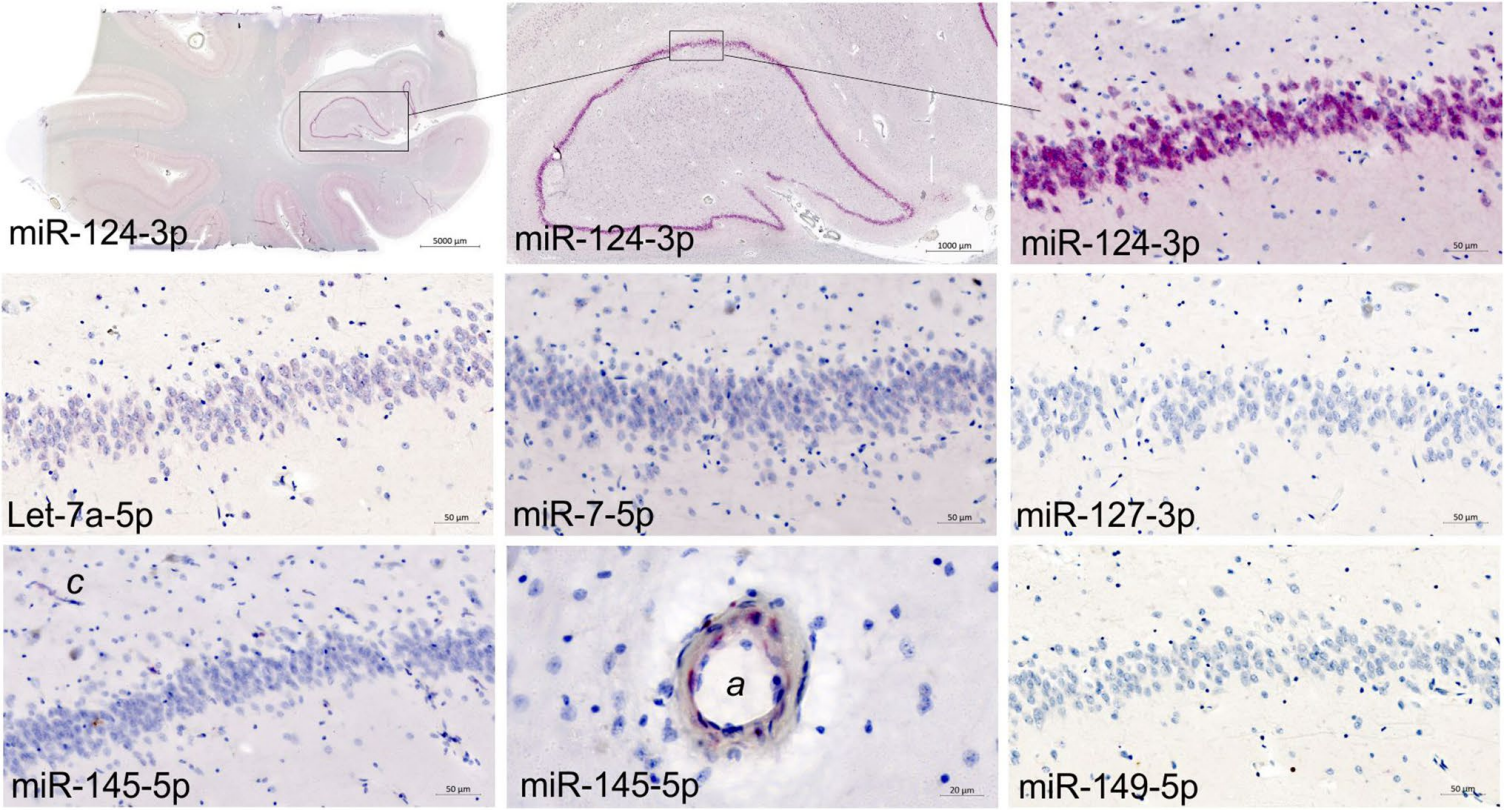
Expression of selected microRNAs in hippocampus investigated using in situ Hybridization (ISH). Tissue sections from hippocampus samples were stained with selected microRNA probes (Table 3) using automated miRNAScope. Discrete diffuse staining is seen with probes to let-7a-5p and miR-7-5p in the dentate granule cell layer. Intense ISH signal is seen with the miR-124-3p probe, whereas no ISH signal is seen with probes to miR-127-3p, miR-145-5p and miR-149-5p in the dentate granule cell layer. The miR-145-5p probe stained capillaries (*c*) and arteries (*a*) in hippocampus samples

In the hippocampus samples we obtained ISH signal with 4 of the 6 selected miRNAscope probes (miR-145-5p, let-7a-5p, miR-124-3p and miR-7-5p). We found the most intense ISH signal from neuronal cells in the pyramidal layers of the CA1-3 and dentate gyrus (DG) with miR-124-3p. ISH signal for let-7a-5p, miR-7-5p and miR-124-3p was seen in the dentate granule cell layer (Fig. 6). The miR-145 ISH signal was noted in vascular structures. No ISH signal was obtained with the probes for miR-127-3p and miR-149-5p (Fig. 6). A manual scoring of all sections was conducted using a binary scale, with present or absent of ISH signal. The results are summarized in Table 3 and ST4.

**Table 3:** Scores from hippocampus with binary staining score (1, 0, NA)

miRNA	Number of sections stained	1	0	NA
let-7a-5p	30	29	0	1
miR-7-5p	30	29	0	1
miR-124-3p	30	29	0	1
miR-127-3p	30	0	29	1
miR-145-5p	30	29	0	1
miR-149-5p	30	0	28	2

## Discussion

The old Brain Collection contains more than 50,000 pieces of post-mortem brain tissue preserved in paraffin blocks. The brains were collected during 1945–1982 from patients with mental disorders not treated with modern medicine. As such, the brain collection represents valuable biological material for potential studies on molecular differences between SCZ, BD, or MDD. In this study, we used nanoString nCounter to identify and select miRNA candidates that we found to be differentially expressed among the selected disorder categories [Kaadt E. Unpublished personal communication], and questioned if the recently developed ISH technology, miRNAscope™ could be applied for in situ detection of the miRNAs in such old samples. Indeed, we found that this was possible, and we assembled a tissue sample cohort from female and male patients diagnosed with one of the three most common mental disorders (SCZ, BD, or MDD) from prefrontal cortex and hippocampus. Six out ten miRNAscope probes selected for staining in prefrontal cortex and four of six probes selected for staining in hippocampus samples were found to provide genuine ISH signal, with the miR-124-3p as expected being the most prevalently expressed. Although the probe design does not allow technical claims on the specificity of the miRNAscope probes, like RNAscope probes for long RNAs have an in-built specificity design [17], the miRNAscope ISH signal observed appears genuine with cell-associated signal and expression in the brain cells. Our findings suggest that the miRNAscope probes can indeed be used for miRNA ISH analyses in the post-mortem collected FFPE samples stored for at present 42–76 years. Hence, the Danish Brain Collection represents a precious biobank not least for miRNA studies, but probably also other molecular analyses.

### Staining Specificity of in situ Hybridization Signal

The automated miRNAscope ISH kit and study design will typically include a probe against snRNA U6 as a positive control probe and a negative control probe, “scramble”. These reference probes appear to be historically valued probes originally introduced in LNA probe-based miRNA ISH analyses [20–22]. The two control probes were only used in the initial phase of our study since they here showed the expected output and because of the following considerations. The two reference probes were indeed valuable to implement the ISH assay in the laboratory, but the snRNA U6 probe may not be the obvious choice as reference probe in miRNA studies. The snRNA U6 probe recognizes a small nuclear RNA (snRNA), and not a miRNA, however, the U6 snRNA has been used in several microarray and quantitative real-time PCR profiling studies for normalization of RNA expression data because of its high expression levels and presence in all tissues [23]. The scramble probe contains a random generic nucleotide sequence, with similar nucleotide design as the specific miRNAscope probes, but with a sequence not being antisense to any human target RNAs. The scramble probe will show potential non-specific reactions caused by the probe itself or the detection reagents. After these two reference probes provided intense positive and clean negative signal, respectively, we considered an endogenously and abundantly expressed miRNA as a better reference as positive control and for qualifying our FFPE samples for their miRNA content. For miRNA ISH studies on brain samples, miR-124-3p has been reported a positive control and reference [20]. We found that the miR-124-3p miRNAscope probe in our sample cohort resulted in ISH signal of varying intensity that allowed us to judge presence or lack of ISH signal obtained with the other weakly expressed miRNAs.

The miRNAscope probes are designed to detect the mature form of miRNAs, but because precursor forms contain the same sequence, the probes cannot discriminate between them. Additionally, for closely related family members with only one or two nucleotide differences, the probes may bind both homologous sequences and, with weaker affinity, to similar family members, making it challenging to achieve perfect specificity.

### RNAscope vs. NanoString Expression Data

The miRNA candidates selected for ISH analyses were partly based on miRNA profiling data, considering prevalence and differential expression among sex and disorder categories, however, not all of the miRNAscope probes resulted in an ISH signal in the samples. E.g., in prefrontal cortex samples, we obtained ISH signal with six out of ten probes, whereas four probes did not show any ISH signal. The lack of ISH signal was surprising since all four microRNAs were detected in the nanoString profiling analysis with adequate expression levels well above background and similar to some of the other detected miRNAs, and therefore the lack of ISH signal was not considered a sensitivity issue. In addition, the miRNAscope technology has been designed with the well-known amplification chemistry known from RNAscope providing single molecule detection sensitivity [17]. We therefore speculate whether the lack of signal can be explained by 1) variation in accessibility or differential degradation, 2) that the sequence detected by nanoString technology is different from that in the miRNAscope probe design, or 3) it may be that the target or the probe itself forms a tertiary structure that prevents the probe from binding to the endogenous target. Future studies may help to clarify the reasons for lack of ISH signal with this subset of probes.

### Consistent Expression Patterns

We found that three of the miRNAscope probes (miR-9-5p, miR-124-3p and miR-138-5p), showed ISH signal in all the 28 samples included after the initial exclusion of two samples. Despite variation in ISH signal intensity, the localization patterns observed were different for the three, but consistent among patients. Importantly, the different expression patterns, miR-9-positive cells being considered astrocytes or microglia and miR-124-3p-positive cell being considered neurons, suggest involvement in different biological processes in the intraneuronal communication. Together, this may be helpful for further biomarker analyses especially in the prefrontal cortex cell layers and in hippocampus, where molecular variation may be linked to various psychiatric disorders, as well as dementia and Alzheimer’s disease [24].

Previously, miR-124-3p has been associated with MDD, SCZ, and BD in postmortem prefrontal cortex human brain samples [11–13], and dysregulated miR-124-3p blood levels has been linked to MDD [11, 14]. We found that miR-9-5p was also abundantly expressed in the brain samples, but the expression pattern was more restricted than miR-124-3p. In a recent study, it was reported that low miR-9-5p levels measured in blood was linked to SCZ [25]. We found miR-138-5p expression in various neurons of the prefrontal cortex layers. miR-138-5p has previously been shown to be expressed in Purkinje cells of the cerebellum [26]. To our knowledge miR-138-5p has not previously been investigated in relation to psychiatric disorders. However, miR-138-5p has been reported to be up-regulated in the cerebrospinal fluid [27], exosomes [28], and hippocampus [29] of patients with Alzheimer´s disease.

The remaining probes that resulted in ISH signal in the prefrontal cortex samples included miR-29b-3p, miR-125b-5p, miR-181a-5p, miR-145-5p, let-7a-5p, and miR-7-5p in hippocampus samples. Common for all of these was that the ISH signal intensity was low or seen only in a few cells compared to miR-9-5p, miR-124-3p and miR-138-5p. Interestingly, both miR-29b-3p and miR-125b-5p measured in blood samples have both been reported to be upregulated in patients with BD compared to the healthy controls [30], and in a comprehensive meta-analysis, Liu et al. found that miR-7-5p and miR-181b-5p, were upregulated in SCZ patients [14]. Maffioletti et al. reported that circulating let-7a-5p was altered in patients with MDD [31]. Thus, these miRNAs may indeed be relevant for further analyses in the respective patient groups, however, finding increased levels of miRNAs in the blood/circulation may not necessarily be caused by a change of expression levels in the brain, but may reflect a systemic imbalance in the patients. Epigenetic modifications are usually tissue-specific, and any study relying on peripheral tissues is unlikely to reflect biological processes in the brain [32, 33]. Not surprisingly, the miR-145-5p ISH signal was seen in capillaries and small arteries in the brain tissue and not in the neuronal cells themselves. Expression of miR-145-5p is associated with vascular smooth muscle cells/pericytes and smooth muscle cells in general [20, 21, 34], and has been reported to be important for the response to oxygen restriction, by carotid artery ligation in MIR145-/- mice and increased in primary rat cortical pericytes under hypoxic conditions [34, 35]. Interestingly, vascularization of brain tissue has been reported to be abnormal/disrupted in both mental and neurodegenerative disorders [36–38]. Thus, even though miR-145-5p is not expressed in neuronal cells, it may serve as an important marker of vascularization in the brain tissue.

## Conclusion

The Danish Brain Collection is unique in the world for several reasons: the high number of patients with psychiatric disorders not treated with modern medicine, the updated diagnostic information, the disease history recorded as well as the decent conditions of the tissue specimens. We showed that miRNAscope ISH can be used to investigate a broad range of miRNAs in both prefrontal cortex and hippocampus specimens. The possibility of being able to measure the presence of miRNA in long-term stored biological material will open new opportunities not only in the biobank material used in this study, but also to a broader extent. Even in pre-historic samples, probably due to their high copy number and protection by protein complexes, miRNAs were found preserved [39].

## Supplementary Information

Below is the link to the electronic supplementary material.

Supplementary file1 (PDF 927 KB)

## Data Availability

No datasets were generated or analysed during the current study.
