# Supplementary material for: Application of microRNA In Situ Hybridization on Long-term Stored Human Formalin-fixed Paraffin-embedded Brain Samples from Psychiatric Patients
Source: Mol Neurobiol. 2025 May 31;62(10):12736–46. doi: 10.1007/s12035-025-05077-z (PMC12433438; doi:10.1007/s12035-025-05077-z)
Supplement: Supplementary file 1 — Supplementary file1 (PDF 927 KB) [file 12035_2025_5077_MOESM1_ESM.pdf]

Supplementary material

Application of microRNA in situ hybridization on long-term stored human formalin-fixed paraffin-embedded brain samples from psychiatric patients

Rolf Søkilde [a], Erik Kaadt [a], Lasse Sommer Kristensen [b], Morten T Venø [c], Jørgen Kjems [d, e], Jens Nyengaard [f, g], Boye Schnack Nielsen [h], Betina Elfving [a]

[a] Experimental and Molecular Psychiatry, Translational Neuropsychiatry Unit, Department of Clinical Medicine, Aarhus University, Palle Juul-Jensens Blvd 11, 8200 Aarhus, Denmark.

[b] The laboratory for Gene-Regulatory Mechanisms in Cancer, Department of Biomedicine, Aarhus University, Høegh-Guldbergs Gade 10, 8000 Aarhus, Denmark

[c] omiics ApS, Åbogade 15, 8200 Aarhus, Denmark

[d] Department of Molecular Biology and Genetics (MBG), Aarhus University, Nørrebrogade 44, 8000 Aarhus, Denmark

[e] Interdisciplinary Nanoscience Center (iNANO), Aarhus University, Gustav Wieds Vej 14, 8000 Aarhus, Denmark

[f] Core Center for Molecular Morphology, Section for Stereology and Microscopy, Department of Clinical Medicine, Aarhus University, Palle Juul-Jensens Blvd 35, 8200 Aarhus, Denmark

[g] Department of Pathology, Aarhus University Hospital, Palle Juul-Jensens Vej 99, 8200 Aarhus, Denmark

[h] Department of Cellular Engineering & Disease Modeling, Bioneer A/S, Kogle Alle 2, 2970 Hørsholm, Denmark

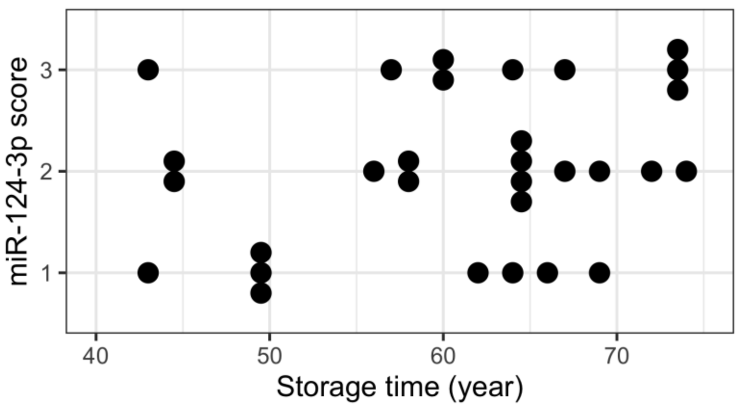

Supplemental Figure 1. miR-124-3p score versus storage time.

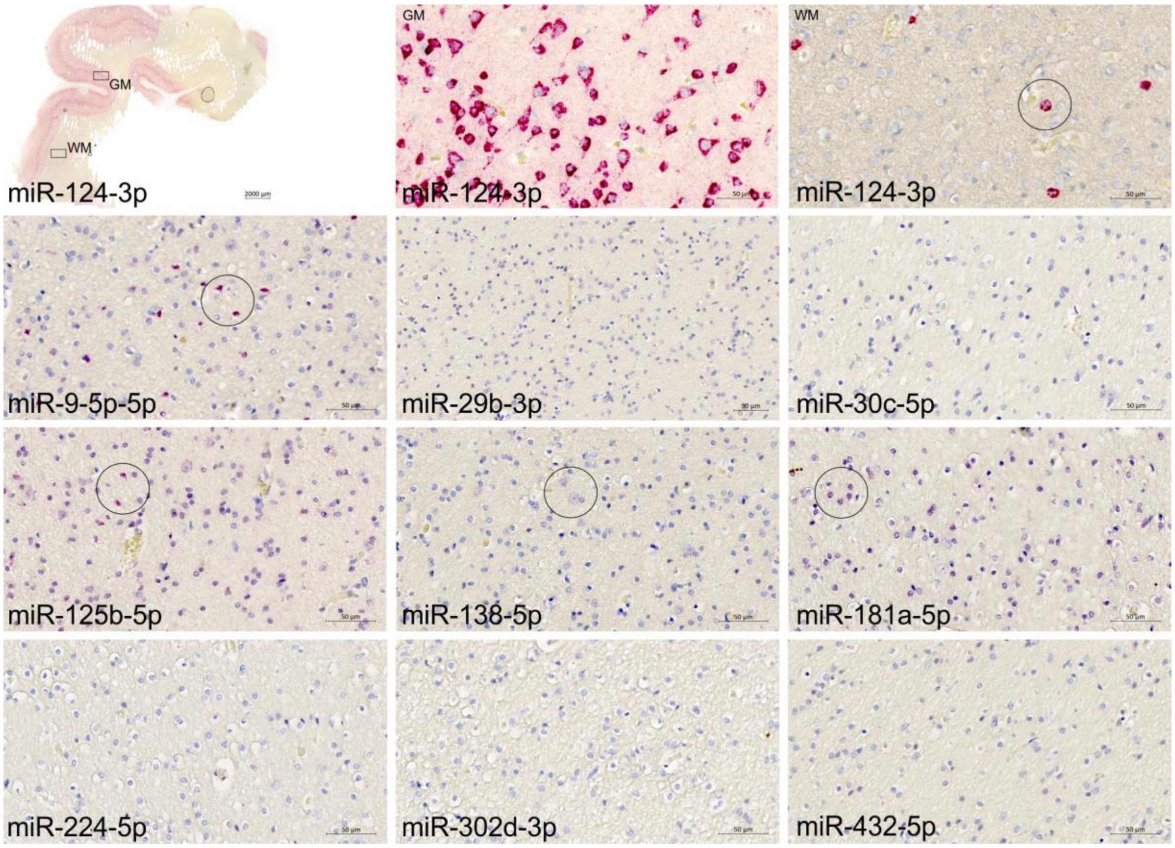

Supplemental Figure 2. Expression of selected microRNAs in prefrontal cortex, white matter. Tissue sections from (an) old prefrontal cortex sample were stained for selected miRNAs (Table 2) using automated miRNAScope. Intense *in situ* Hybridization (ISH) signal is seen in the grey matter (GM) with the miR-124 probe (upper row, middle), whereas very few positive cells are found in white matter (WM, upper row right). Probes for the other miRNAs show a discrete or no signal. Positive ISH signal (examples indicated by circles) is seen with probes against miR-9-5p, miR-125b-5p, miR-138-5p and miR-181a-5p, whereas no ISH signal is seen with probes to miR-29b-3p, miR-30c-5p, miR-224-5p, miR-302d-3p and miR-432-5p.

ST1_nanostring_cohort

sampleID	sampleID from the braincollection	
sex	sex of the individual	Male/Female
diagnosis_short	the diagnosis of the individual	MDD/SCZ/BD
PFC_nanostring	has this individual contributed a sample to nanostring expression profiling prefrontal cortex	1/0
HIP_nanostring	has this individual contributed a sample to nanostring expression profiling hippocampus	1/0
PFC_ISH	has this individual contributed a sample to ISH prefrontal cortex	1/0
HIP_ISH	has this individual contributed a sample to ISH hippocampus	1/0

ST2_probe_overview

column name	column description
probe type	RNA class targeted på probe
short name	short name of miRNAs without the species prefix
microRNA	miRBase ver 22.1 microRNA name
mirbase id	miRBase ver 22.1 microRNA id
ACD probe	probe name in ACD homepage
Prefrontal cortex	is the probe used for prefrontal cortex samples (1,0)
Hippocampus	is the probe used for hippocampus samples (1,0)

ST3_summary_stats_samples

sex	sex of the individual	Male/Female
diagnosis_short	the diagnosis of the individual	
n	number of samples in category	
mean_age_at_death	mean age of the individual at the time of death	years

ST4_cortex_results

binary scores for all probes in prefrontal cortex samples and semi quantitative scale (1,2,3) for miR-124-3p

ST5_hippocampus_results

binary scores for all probes in hippocampus samples

SUPPLEMENTARY TABLE 1_nanoString _ISH_cohort

Sample ID	Time of death (year)	Sex	Diagnosis	Age at death	Storage time (year)	PFC_nanostring	HIP_nanostring	PFC_ISH	HIP_ISH
1	1947	Male	MDD	47	76	0	1	0	1
2	1948	Female	SCZ	61	75	1	0	0	0
3	1948	Male	BD	56	75	1	1	0	1
4	1949	Female	MDD	51	74	1	0	0	0
5	1949	Female	SCZ	54	74	1	0	1	0
6	1949	Female	MDD	49	74	1	1	1	0
7	1949	Female	BD	45	74	1	0	1	0
8	1950	Female	SCZ	74	73	1	0	0	0
9	1950	Female	BD	70	73	1	0	0	0
10	1950	Female	SCZ	50	73	1	1	0	0
11	1950	Female	MDD	46	73	1	0	1	0
12	1950	Male	SCZ	57	73	0	1	0	0
13	1950	Male	BD	60	73	0	1	0	0
14	1951	Female	BD	64	72	1	0	0	0
15	1951	Male	BD	56	72	1	0	0	0
16	1951	Female	BD	77	72	1	0	1	0
17	1951	Female	SCZ	42	72	1	0	0	0
18	1951	Female	BD	78	72	1	0	0	0
19	1951	Male	MDD	62	72	0	1	0	0
20	1952	Female	SCZ	73	71	0	1	0	0
21	1952	Female	MDD	47	71	0	1	0	0
22	1952	Female	MDD	50	71	0	1	0	1
23	1953	Female	MDD	70	70	1	1	0	1
24	1953	Female	BD	82	70	1	1	0	1
25	1953	Female	MDD	82	70	0	1	0	0
26	1953	Male	MDD	37	70	1	1	0	0
27	1953	Female	MDD	56	70	1	0	0	0
28	1953	Male	SCZ	58	70	1	1	0	0
29	1953	Male	BD	49	70	1	1	0	0
30	1954	Female	BD	52	69	0	1	0	0
31	1954	Male	MDD	48	69	1	0	0	0
32	1954	Male	MDD	64	69	1	1	1	0
33	1954	Male	SCZ	60	69	1	1	1	1
34	1954	Male	BD	61	69	1	0	0	0
35	1954	Female	BD	60	69	1	1	0	0
36	1955	Female	BD	85	68	1	1	0	0
37	1955	Female	SCZ	72	68	1	1	0	0
38	1955	Female	SCZ	54	68	1	1	0	0
39	1955	Female	SCZ	77	68	1	0	0	0
40	1955	Female	MDD	76	68	0	1	0	0
41	1956	Female	MDD	50	67	1	0	1	0
42	1956	Male	SCZ	63	67	0	1	0	1
43	1956	Female	BD	50	67	1	1	0	1
44	1956	Female	SCZ	61	67	1	1	1	1
45	1956	Female	BD	72	67	1	1	0	1
46	1956	Female	BD	63	67	1	0	0	0
47	1956	Male	MDD	unknown	67	0	1	0	1
48	1957	Female	BD	83	66	1	1	1	0
49	1957	Female	MDD	84	66	1	1	0	1
50	1957	Female	SCZ	74	66	1	1	0	1
51	1957	Female	BD	72	66	1	0	0	0
52	1957	Female	BD	83	66	1	0	0	0
53	1957	Male	SCZ	65	66	1	1	0	1
54	1957	Female	SCZ	68	66	1	0	0	0
55	1957	Female	SCZ	66	66	0	1	0	0
56	1957	Female	BD	unknown	66	1	1	0	0
57	1957	Female	SCZ	68	66	1	1	0	1
58	1958	Female	BD	50	65	1	0	1	0
59	1958	Male	SCZ	51	65	0	1	0	0
60	1958	Female	SCZ	61	65	1	1	1	0
61	1958	Female	BD	70	65	1	1	0	0
62	1958	Female	SCZ	70	65	1	1	0	0
63	1958	Male	BD	48	65	1	1	1	0
64	1958	Male	SCZ	59	65	1	0	0	0
65	1958	Female	BD	48	65	0	1	0	0
66	1958	Male	MDD	47	65	1	0	0	0
67	1958	Female	BD	85	65	1	1	0	0
68	1959	Female	SCZ	72	64	1	1	1	1
69	1959	Male	MDD	65	64	1	1	0	0
70	1959	Female	SCZ	54	64	1	0	0	0
71	1959	Female	BD	80	64	1	0	0	0
72	1959	Female	BD	81	64	1	1	1	0
73	1959	Male	SCZ	64	64	1	0	0	0
74	1959	Male	BD	63	64	1	1	1	1
75	1959	Female	SCZ	69	64	1	1	0	0
76	1959	Female	BD	53	64	0	1	0	0
77	1959	Female	SCZ	77	64	1	1	0	0
78	1959	Male	MDD	63	64	0	1	0	0
79	1959	Male	BD	58	64	1	0	0	0
80	1959	Male	BD	50	64	1	0	0	0
81	1959	Female	BD	72	64	1	1	0	1

82	1959	Female	BD	68	64	0	1	0	0
83	1960	Female	SCZ	68	63	1	1	0	0
84	1960	Female	MDD	74	63	0	1	0	0
85	1960	Female	BD	83	63	1	0	0	0
86	1961	Male	BD	60	62	1	0	0	0
87	1961	Female	MDD	47	62	1	1	0	0
88	1961	Female	SCZ	88	62	1	1	1	0
89	1961	Female	BD	74	62	0	1	0	0
90	1961	Female	MDD	72	62	1	1	0	0
91	1961	Female	BD	65	62	1	1	0	0
92	1961	Female	MDD	69	62	0	1	0	0
93	1962	Female	SCZ	54	61	0	1	0	0
94	1962	Male	BD	61	61	0	1	0	0
95	1962	Female	SCZ	63	61	0	1	0	0
96	1962	Female	SCZ	73	61	0	1	0	0
97	1962	Male	BD	63	61	1	1	0	0
98	1962	Female	MDD	69	61	0	1	0	0
99	1963	Male	MDD	65	60	0	1	0	0
100	1963	Female	BD	72	60	1	1	0	0
101	1963	Female	MDD	77	60	1	1	1	1
102	1963	Male	MDD	50	60	1	0	1	0
103	1963	Female	MDD	74	60	0	1	0	0
104	1963	Male	SCZ	56	60	0	1	0	0
105	1963	Female	MDD	75	60	1	1	0	0
106	1963	Male	BD	50	60	1	1	0	0
107	1964	Female	SCZ	63	59	0	1	0	0
108	1964	Female	SCZ	70	59	1	0	0	0
109	1964	Male	SCZ	65	59	1	0	0	0
110	1964	Male	MDD	58	59	1	0	0	0
111	1964	Female	BD	74	59	1	1	0	1
112	1964	Male	BD	56	59	1	1	0	0
113	1965	Female	SCZ	74	58	0	1	0	1
114	1965	Male	BD	59	58	0	1	0	0
115	1965	Female	MDD	79	58	0	1	0	0
116	1965	Female	MDD	73	58	0	1	0	0
117	1965	Male	SCZ	52	58	1	1	1	1
118	1965	Male	MDD	56	58	1	1	1	0
119	1966	Male	BD	63	57	0	1	0	0
120	1966	Male	MDD	47	57	1	1	0	0
121	1966	Male	MDD	62	57	1	1	0	0
122	1966	Female	MDD	62	57	1	1	1	0
123	1967	Male	MDD	60	56	1	1	1	0
124	1968	Male	BD	65	55	0	1	0	1
125	1968	Male	SCZ	64	55	1	0	0	0
126	1969	Male	BD	64	54	1	1	0	0
127	1969	Male	SCZ	53	54	0	1	0	0
128	1969	Male	SCZ	56	54	1	1	0	0
129	1969	Male	BD	55	54	1	1	0	0
130	1969	Female	SCZ	65	54	0	1	0	0
131	1969	Male	SCZ	62	54	1	0	0	0
132	1969	Female	MDD	58	54	0	1	0	0
133	1969	Female	MDD	87	54	1	1	0	1
134	1970	Male	MDD	60	53	1	1	0	1
135	1970	Male	SCZ	59	53	1	1	0	0
136	1971	Male	MDD	57	52	0	1	0	0
137	1971	Male	MDD	63	52	1	1	0	0
138	1972	Female	BD	90	51	1	1	0	0
139	1972	Male	MDD	50	51	1	1	0	0
140	1972	Female	MDD	86	51	0	1	0	0
141	1972	Male	SCZ	63	51	1	1	0	0
142	1972	Male	SCZ	64	51	1	1	0	1
143	1973	Male	BD	51	50	1	1	0	1
144	1973	Male	SCZ	56	50	1	1	1	0
145	1973	Male	SCZ	62	50	1	1	0	0
146	1973	Male	SCZ	53	50	0	1	0	0
147	1974	Male	MDD	53	49	1	1	1	1
148	1974	Male	BD	62	49	1	0	1	0
149	1974	Male	SCZ	51	49	1	1	0	0
150	1974	Male	BD	65	49	0	1	0	1
151	1974	Male	MDD	64	49	1	1	0	0
152	1974	Male	MDD	54	49	0	1	0	0
153	1976	Male	SCZ	63	47	0	1	0	0
154	1976	Male	BD	56	47	0	1	0	0
155	1977	Female	MDD	60	46	1	0	0	0
156	1977	Male	SCZ	61	46	1	0	0	0
157	1978	Male	BD	64	45	1	0	1	0
158	1978	Male	MDD	64	45	1	1	0	0
159	1979	Male	SCZ	64	44	1	0	1	0
160	1979	Male	SCZ	58	44	1	0	0	0
161	1980	Male	SCZ	51	43	1	0	1	0
162	1980	Male	BD	45	43	1	1	1	0
163	1981	Male	MDD	71	42	1	1	0	1

sum	117	119	30	30

SUPPLEMENTARY TABLE 2_Probe overview

probe type	short name	microRNA	mirbase id	ACD probe	Prefrontal cortex	Hippocampus	lowest calculated p-value
miRNA	let-7a-5p	hsa-let-7a-5p	MIMAT0000062	SR-hsa-let-7a-5p-S1	0	1	2.06E-04
miRNA	miR-7-5p	hsa-miR-7-5p	MIMAT0000252	SR-hsa-miR-7-5p-S1	0	1	1.55E-02
miRNA	miR-9-5p	hsa-miR-9-5p	MIMAT0000441	SR-hsa-miR-9-5p-S1	1	0	6.69E-04
miRNA	miR-29b-3p	hsa-miR-29b-3p	MIMAT0000100	SR-hsa-miR-29b-3p-S1	1	0	6.57E-03
miRNA	miR-30c-5p	hsa-miR-30c-5p	MIMAT0000244	SR-hsa-miR-30c-5p-S1	1	0	1.76E-02
miRNA	miR-124-3p	hsa-miR-124-3p	MIMAT0000422	SR-hsa-miR-124-3p-S1	1	1	6.73E-03
miRNA	miR-125b-5p	hsa-miR-125b-5p	MIMAT0000443	SR-hsa-miR-125b-5p-S1	1	0	5.86E-03
miRNA	miR-127-3p	hsa-miR-127-3p	MIMAT0000446	SR-hsa-miR-127-3p-S1	0	1	2.33E-02
miRNA	miR-138-5p	hsa-miR-138-5p	MIMAT0000430	SR-hsa-miR-138-5p-S1	1	0	9.10E-03
miRNA	miR-145-5p	hsa-miR-145-5p	MIMAT0000437	SR-hsa-miR-145-5p-S1	0	1	1.46E-04
miRNA	miR-149-5p	hsa-miR-149-5p	MIMAT0000450	SR-hsa-miR-149-5p-S1	0	1	8.77E-03
miRNA	miR-181a-5p	hsa-miR-181a-5p	MIMAT0000256	SR-hsa-miR-181a-5p-S1	1	0	3.41E-02
miRNA	miR-224-5p	hsa-miR-224-5p	MIMAT0000281	SR-hsa-miR-224-5p-S1	1	0	2.31E-03
miRNA	miR-302d-3p	hsa-miR-302d-3p	MIMAT0000718	SR-hsa-miR-302d-3p-S1	1	0	3.22E-05
miRNA	miR-432-5p	hsa-miR-432-5p	MIMAT0002814	SR-hsa-miR-432-5p-S1	1	0	1.25E-02
positive control	NA	NA	NA	SR-RNU6-S1	1	1	
negative control	NA	NA	NA	SR-Scramble-S1	1	1	

SUPPLEMENTARY TABLE 3_Prefrontal cortex results

Sample ID	Age at death	Storage time (year)	miR-124-3p		miR-124-3p	miR-9-5p	miR-29b-3p	miR-125b-5p	miR-138-5p	miR-432-5p	miR-302d-3p	miR-224-5p	miR-30c-5p	miR-181a-5p	general note
5	54	74	3		1	1	1	1	1	0	0	0	0	1	
6	49	74	2		1	1	1	1	1	0	0	0	0	1	
7	45	74	3		1	1	1	1	1	0	0	0	0	1	
11	46	73	3		1	1	1	1	1	0	0	0	0	1	
16	77	72	2		1	1	1	1	1	0	0	0	0	1	
32	64	69	1		low	low	0	NA	NA	NA	NA	NA	NA	NA	low expression sample, was taken out to make room for positive control
33	60	69	2		1	1	0	1	1	0	0	0	0	1	
41	50	67	3		1	1	1	1	1	0	0	0	0	1	
44	61	67	2		1	1	1	1	1	0	0	0	0	1	
48	83	66	1		1	1	0	1	1	0	0	0	0	1	
58	50	65	2		1	1	0	1	1	0	0	0	0	1	
60	61	65	2		1	1	1	1	1	0	NA*	0	0	1	
63	48	65	2		1	1	1	1	1	0	NA*	0	0	NA poor section	
68	72	64	3		1	1	1	1	1	0	0	0	0	1	
72	81	64	2		1	1	0	1	1	0	0	0	0	1	
74	63	64	1		low	low	0	NA	NA	NA	NA	NA	NA	NA	low expression sample, was taken out to make room for positive control
88	88	62	1		1	1	1	1	1	0	0	0	0	1	
101	77	60	3		1	1	1	1	1	0	0	0	0	1	
102	50	60	3		1	1	1	1	1	0	0	0	0	1	
117	52	58	2		1	1	1	1	1	0	0	0	0	1	
118	56	58	2		1	1	0	1	1	0	0	0	0	1	
122	62	57	3		1	1	1	1	1	0	0	0	0	1	
123	60	56	2		1	1	1	1	1	0	0	0	0	1	
144	56	50	1		1	1	0	1	1	0	0	0	0	1	
147	53	49	1		1	1	0	1	1	0	0	0	0	1	
148	62	49	1		1	1	0	1	1	0	0	0	0	1	
157	64	45	2		1	1	1	1	1	0	0	0	0	1	
159	64	44	2		1	1	1	1	1	0	0	0	0	1	
161	51	43	1		1	1	0	1	1	0	0	0	0	1	
162	45	43	3		1	1	0	1	1	0	0	0	0	1	

*broken slide

SUPPLEMENTARY TABLE 4_Hippocampus results Binary scores

sample ID	miR-124-3p	let-7a-5p	miR-127-3p	miR-145-5p	miR-7-5p	miR-149-5p	general note on samples
1	1	1	0	1	1	0	
3	1	1	0	1	1	0	
22	1	1	0	1	1	0	
23	1	1	0	1	1	0	
24	1	1	0	1	1	0	
33	1	1	0	1	1	0	
42	1	1	0	1	1	0	No Hippocampus
43	1	1	0	1	1	0	
44	1	1	0	1	1	0	
45	1	1	0	1	1	0	
47	NA	NA	NA	NA	NA	NA	poor tissue adherence
49	1	1	0	1	1	NA*	
50	1	1	0	1	1	0	No Hippocampus
53	1	1	0	1	1	0	
57	1	1	0	1	1	0	
68	1	1	0	1	1	0	poor tissue morpholy
74	1	1	0	1	1	0	
81	1	1	0	1	1	0	
101	1	1	0	1	1	0	
111	1	1	0	1	1	0	
113	1	1	0	1	1	0	
117	1	1	0	1	1	0	
124	1	1	0	1	1	0	
133	1	1	0	1	1	0	
134	1	1	0	1	1	0	
142	1	1	0	1	1	0	
143	1	1	0	1	1	0	No Hippocampus
147	1	1	0	1	1	0	No Hippocampus
150	1	1	0	1	1	0	
163	1	1	0	1	1	0	

*broken slide

SUPPLEMENTARY TABLE 5_Summary_stats_samples

Sex	Diagnosis	n	Mean age at death	
Female	BD	31	67.8	66.7
Female	MDD	26	66.3	
Female	SCZ	29	66.0	
Male	BD	25	57.6	57.1
Male	MDD	25	54.7	
Male	SCZ	27	58.9	
